# Pathological Complete Response to a Single Dose of Pembrolizumab‐Based Chemoimmunotherapy for Squamous Cell Carcinoma of the Lung: A Case Report

**DOI:** 10.1111/1759-7714.15519

**Published:** 2024-12-17

**Authors:** Yugo Matsumura, Seiya Ichihara, Kaori Nii, Kazumasa Nanjo, Naoki Kadota, Yoshio Okano, Hisanori Machida, Nobuo Hatakeyama, Hiroyuki Hino, Keishi Naruse, Tsutomu Shinohara, Shoji Sakiyama, Eiji Takeuchi

**Affiliations:** ^1^ Department of Respiratory Medicine National Hospital Organization Kochi Hospital Kochi City Kochi Japan; ^2^ Department of Thoracic Surgery National Hospital Organization Kochi Hospital Kochi City Kochi Japan; ^3^ Department of Pathology National Hospital Organization Kochi Hospital Kochi City Kochi Japan; ^4^ Department of Community Medicine for Respirology, Graduate School of Biomedical Sciences Tokushima University Tokushima Japan; ^5^ Department of Clinical Investigation National Hospital Organization Kochi Hospital Kochi City Kochi Japan

**Keywords:** immune checkpoint inhibitors, non‐small‐cell lung cancer, pathological complete response, single dose, squamous cell carcinoma

## Abstract

We herein describe a patient with non‐small‐cell lung cancer who achieved pCR with a single dose of pembrolizumab‐based chemoimmunotherapy followed by surgery. A 61‐year‐old man was referred to our hospital with wheezing and an abnormal chest shadow. Squamous cell carcinoma of the left lower lobe, cT2aN1M0 stage IIB, was diagnosed and pembrolizumab‐based chemoimmunotherapy was initiated at the patient's request. One month later, chest CT revealed new ground‐glass opacities of the lungs, which were judged to be a CTCAE grade 2 pneumonitis due to an immune‐related adverse event (irAE). Therefore, steroid therapy was initiated. Prednisolone was tapered and discontinued as symptoms improved. A sleeve resection of the left lower lobe was performed, and a pathological complete response (pCR) was confirmed in a resected specimen. There has been no recurrence for 1 year and 7 months without treatment. This is the first case report of pCR to a single dose of chemoimmunotherapy followed by surgery for lung cancer. The present results suggest the potential of a single dose of chemoimmunotherapy to achieve pCR and cause irAEs in some patients.

## Graphical abstract

Clinical course of the patient. Only one cycle of pembrolizumab‐based chemoimmunotherapy was administered. At the end of the first cycle, the patient developed CTCAE grade 2 immune‐related pneumonitis, which necessitated the discontinuation of chemoimmunotherapy. Prednisolone (60 mg/body) was then initiated. Ground‐glass opacities disappeared and, thus, prednisolone was tapered and discontinued. Since there was no metastasis, sleeve resection of the left lower lobe was performed. Pathological findings showed pCR. There has been no recurrence for 1 year and 7 months without treatment.

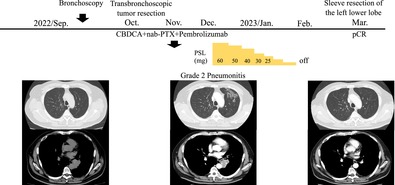

## 1 Introduction

Lung cancer treatment has been revolutionized by the advent of immune checkpoint inhibitors (ICIs), such as pembrolizumab. In patients with previously untreated metastatic squamous non‐small cell lung cancer (NSCLC), the addition of pembrolizumab to carboplatin plus paclitaxel or nab‐paclitaxel therapy led to significantly longer overall survival and progression‐free survival than chemotherapy alone (KEYNOTE 407) [1]. Pembrolizumab, carboplatin, and nab‐paclitaxel are standard first‐line treatments for unresectable squamous NSCLC.

In patients with advanced NSCLC, the combination of nivolumab and chemotherapy for three cycles before surgery provided significant benefits for event‐free survival and pathological complete response (pCR) over chemotherapy alone (CheckMate 816) [2]. Four phase 3 trials (neoadjuvant and perioperative approaches to lung cancer) were recently published and reported a lower risk of recurrence [3, 4, 5, 6]. However, the development of serious immune‐related adverse events (irAEs) necessitated the discontinuation of treatment in some cases. Nevertheless, a single dose of chemoimmunotherapy for lung cancer rarely results in pCR.

We herein describe a patient with NSCLC who achieved pCR to a single dose of pembrolizumab‐based chemoimmunotherapy followed by surgery.

## 2 Case Report

**FIGURE 1:**
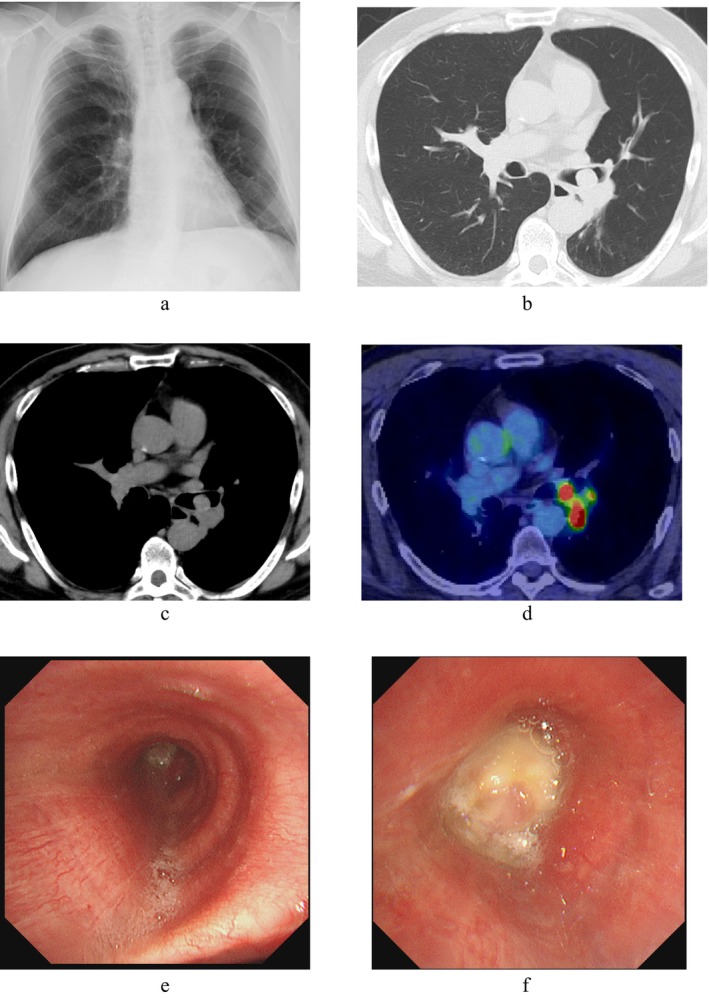
(a) Chest x‐ray, (b, c) computed tomography, and (d) ^18^F‐fluorodeoxyglucose (FDG)‐positron emission tomography/computed tomography (PET/CT) at first admission showed tumors in the left main bronchus and left hilar lymphadenopathy. (e) Bronchoscopy with standard observations from the carina revealed a tumor in front of the left second carina. (f) Bronchoscopy with standard observations showed a tumor obstructing the left main bronchus.

**FIGURE 2:**
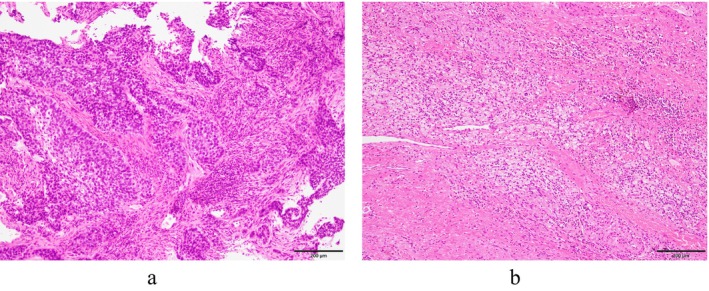
(a) Hematoxylin and eosin‐stained section of squamous cell carcinoma (bar = 200 μm). (b) No residual cancer cells were detected in the resected left lower lobe, and a pathological complete response was diagnosed.

**FIGURE 3:**
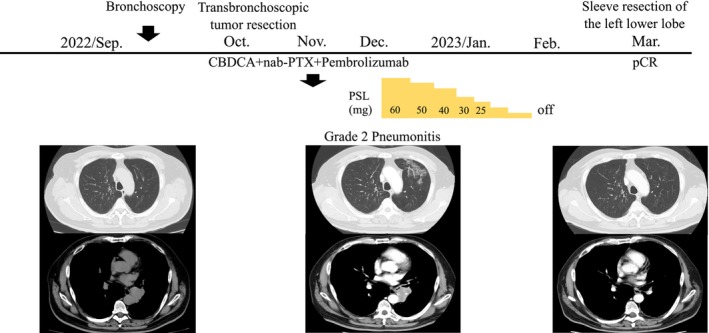
Clinical course of the patient. Only one cycle of pembrolizumab‐based chemoimmunotherapy was administered. At the end of the first cycle, the patient developed CTCAE grade 2 immune‐related pneumonitis, which necessitated the discontinuation of chemoimmunotherapy. Prednisolone (60 mg/body) was then initiated. Ground‐glass opacities disappeared and, thus, prednisolone was tapered and discontinued. CBDCA, carboplatin; nab‐PTX, nanoparticle albumin‐bound paclitaxel; pCR, pathological complete response.

A 61‐year‐old man was referred to our hospital with wheezing and an abnormal chest shadow. The patient had a smoking habit of two packs of cigarettes per day for 42 years. He had COPD, but no concomitant clinical or subclinical autoimmune diseases. The Eastern Cooperative Oncology Group performance status score was 1. Blood tests showed normal ranges for white blood cells (5250/μL), red blood cells (458 × 10^4^/μL), platelets (25.9 × 10^4^/μL), carcinoembryonic antigen (1.2 ng/mL), cytokeratin 19 fragments (1.7 ng/mL), and pro‐gastrin‐releasing peptide (54 pg/mL). The neutrophil‐to‐lymphocyte ratio was 3.0. Chest x‐ray (Figure 1a), computed tomography (CT) (Figure 1b,c), and ^18^F‐fluorodeoxyglucose (FDG)‐positron emission tomography/CT (PET/CT) (Figure 1d) revealed tumors in the left main bronchus and left hilar lymphadenopathy. Bronchoscopy showed that the left main bronchus was obstructed by one of the tumors (Figure 1e,f). Squamous cell carcinoma was diagnosed from transbronchial lung biopsy specimens (Figure 2a). Immunostaining revealed that tumor cells diffusely expressed p40 and were negative for TTF‐1. Immunostaining also showed that more than 85% of tumor cells expressed PD‐L1 (22C3 clones). There were no other sites of distant metastases. Based on these findings, a clinical diagnosis of left lower lobe squamous cell carcinoma cT2aN1M0, stage IIB with no targetable driver mutations was made. Although we proposed radical surgery based on the location of the lesion, the patient refused to undergo left total pneumonectomy. At the patient's request, transbronchoscopic resection of the tumor followed by pembrolizumab‐based chemoimmunotherapy (carboplatin [area under the plasma concentration‐time curve 6, day 1] and nanoparticle albumin‐bound paclitaxel [100 mg/m^2^, days 1, 8, and 15] plus pembrolizumab [200 mg/body, day 1]) was initiated as the first‐line treatment. One month later, chest CT revealed new ground‐glass opacities of the lungs. The patient was clinically diagnosed with common terminology criteria for adverse events grade‐2 immune‐related pneumonitis. Pembrolizumab‐based chemoimmunotherapy was stopped and prednisolone (60 mg/body) was initiated. Ground‐glass opacities subsequently disappeared; therefore, prednisolone was tapered and discontinued (Figure 3). Cancer cells had significantly shrunk. Since there was no metastasis, sleeve resection of the left lower lobe was performed. Pathological findings showed pCR (Figure 2b). There has been no recurrence for 1 year and 7 months without treatment.

## 3 Discussion

We herein described a patient with squamous NSCLC who achieved pCR to a single dose of pembrolizumab‐based chemoimmunotherapy followed by surgery.

To the best of our knowledge, this is the first case report of pCR in lung cancer after surgery following a single dose of chemoimmunotherapy. Previous studies reported profound and durable responses to a single dose of ICI for lung cancer (Table 1) [7, 8, 9, 10]. All patients were male, most had a smoking history, and PD‐L1 expression was high. In each case, the development of severe irAEs necessitated the discontinuation of treatment after one cycle. irAEs were immune‐related pneumonitis in the present case, a myasthenic crisis in the first case [7], pneumonitis in the second and third cases [8, 9], and hepatitis in the fourth case [10]. Similar findings were reported in patients with other cancers [11, 12, 13]. Therefore, even a single dose of chemoimmunotherapy may achieve pCR and cause irAEs. On the other hand, irAEs are mainly attributed to the bystander activation of T‐cells. A previous study suggested that autoimmune toxicities were more likely to develop in patients who responded to ICIs [14].

**TABLE 1:** Profound and durable responses to a single dose of ICIs for lung cancer.

No.	Author	Age (years)	Sex	Smoking history (pack‐years)	Histology	PD‐L1 TPS (%)	PFS (months)	ICIs	Response
1	Tan, Toh, and Takano [7]	45	Male	1	Sq	Unknown	6+	Nivolumab	PR
2	Tokuyasu et al. [8]	73	Male	53	Pleomorphic	100	17+	Pembrolizumab	PR
3	Kato et al. [9]	88	Male	Unknown	Adeno	70	18+	Pembrolizumab	Metabolic
4	Kondo et al. [10]	79	Male	80	Adeno	100	18+	Pembrolizumab	PR
5	The present case	61	Male	84	Sq	85	19+	Pembrolizumab	pCR

Abbreviations: +, continuing; Adeno, adenocarcinoma; ICIs, immune checkpoint inhibitors; pCR, pathological complete response; PD‐L1, programmed death ligand‐1; PFS, progression‐free survival; Pleomorphic, pleomorphic carcinoma; PR, partial response; Sq, squamous cell carcinoma; TPS, tumor proportion score.

As a limitation, it remains unclear whether this effect was due to chemotherapy or immunotherapy. However, the addition of immunotherapy to neoadjuvant chemotherapy significantly increased pCR [2]. Long‐term survivors have also been reported with a single dose of immunotherapy [7, 8, 9, 10]. In addition, this was a single case report. Therefore, further investigations are warranted to establish whether short‐cycle neoadjuvant chemoimmunotherapy is feasible.

In the present case, a single dose of pembrolizumab‐based chemoimmunotherapy avoided total pneumonectomy, resulted in pCR, and there has been no recurrence for 1 year and 7 months; therefore, the effects of immunotherapy may last longer with just one dose. Further studies are needed to identify predictive biomarkers of profound and durable responses to ICIs.

## 4 Conclusion

We herein described a patient with squamous NSCLC who achieved pCR to a single dose of pembrolizumab‐based chemoimmunotherapy followed by surgery. The present results suggest the potential of a single dose of pembrolizumab‐based chemoimmunotherapy alone to achieve pCR and cause irAEs in some patients.

## Author Contributions

**Yugo Matsumura:** conceptualization, methodology, writing – original draft. **Seiya Ichihara:** conceptualization, visualization, resources. **Kaori Nii:** visualization, resources. **Kazumasa Nanjo:** visualization, resources. **Naoki Kadota**, **Yoshio Okano**, **Hisanori Machida**, **Nobuo Hatakeyama**, and **Keishi Naruse:** visualization, investigation. **Hiroyuki Hino:** visualization, resources. **Tsutomu Shinohara:** supervision. **Shoji Sakiyama:** visualization, resources. **Eiji Takeuchi:** conceptualization, methodology, writing – reviewing and editing.

## Ethics Statement

Informed consent was obtained to publish this report.

## Consent

Written informed consent was obtained from the patient to publish anonymized data and accompanying images.

## Conflicts of Interest

The authors declare no conflicts of interest.

## Data Availability

Data that support the present results are available from the corresponding author upon reasonable request.
